# The Return of Blood to the Heart

**Published:** 1991-12

**Authors:** M. G. Wilson


### THE RETURN OF BLOOD TO THE HEART
**Venus pumps in Health and disease**
A. M. N. Gardner and R. H. Fox
John Libbey and Company, London. Price £32

The authors were the first to describe the existence of a "foot pump" which they reported in our predecessor, the Bristol Medico-Chirurgical Journal in 1983. This book is not only a complete statement and elaboration of this discovery but also a review of the entire subject of venous return. In the sole of the foot there are large plantar veins which are emptied by weight bearing and the authors were the first to recognise that this "foot pump" was the means by which much of the venous return from the leg was returned against gravity to the heart. It depended upon the pressure of the weight of the body and not upon muscular action as in the case of the "calf pump" already recognised.

This book however also describes the other mechanisms by which blood is returned to the heart from other parts of the body, the methods by which they have been investigated, their pathology and how the new knowledge can be used in the prophylaxis of deep venous thrombosis and pulmonary embolism. There is a good historical introduction describing the development of the understanding of the circulation from earliest times. There is a bibliography of 588 references and a very complete index. The book is copiously and beautifully illustrated. It brings up to date the knowledge of a subject of great importance in the prevention of pathology and mortality only too commonly the result of venous disease.

M. G. Wilson

